# IL-21 induces antiviral microRNA-29 in CD4 T cells to limit HIV-1 infection

**DOI:** 10.1038/ncomms8562

**Published:** 2015-06-25

**Authors:** Stanley Adoro, Juan R. Cubillos-Ruiz, Xi Chen, Maud Deruaz, Vladimir D. Vrbanac, Minkyung Song, Suna Park, Thomas T. Murooka, Timothy E. Dudek, Andrew D. Luster, Andrew M. Tager, Hendrik Streeck, Brittany Bowman, Bruce D. Walker, Douglas S. Kwon, Vanja Lazarevic, Laurie H. Glimcher

**Affiliations:** 1 Department of Medicine, Weill Cornell Medical College, Cornell University, 1300 York Avenue, New York, New York 10065, USA; 2 Ragon Institute of MGH, MIT and Harvard, Cambridge, Massachusetts 02139, USA; 3 Center for Immunology and Inflammatory Diseases, Division of Rheumatology, Allergy and Immunology, Massachusetts General Hospital, Charlestown, Massachusetts 02129, USA; 4 Institute for Medical Biology, University Hospital Essen, University of Duisburg-Essen, Essen D-45147, Germany; 5 Howard Hughes Medical Institute, Chevy Chase, Maryland 20814, USA; 6 Experimental Immunology Branch, National Cancer Institute, NIH, Bethesda, Maryland 20892, USA

## Abstract

Initial events after exposure determine HIV-1 disease progression, underscoring a critical need to understand host mechanisms that interfere with initial viral replication. Although associated with chronic HIV-1 control, it is not known whether interleukin-21 (IL-21) contributes to early HIV-1 immunity. Here we take advantage of tractable primary human lymphoid organ aggregate cultures to show that IL-21 directly suppresses HIV-1 replication, and identify microRNA-29 (miR-29) as an antiviral factor induced by IL-21 in CD4 T cells. IL-21 promotes transcription of all miR-29 species through STAT3, whose binding to putative regulatory regions within the *MIR29* gene is enriched by IL-21 signalling. Notably, exogenous IL-21 limits early HIV-1 infection in humanized mice, and lower viremia *in vivo* is associated with higher miR-29 expression. Together, these findings reveal a novel antiviral IL-21-miR-29 axis that promotes CD4 T-cell-intrinsic resistance to HIV-1 infection, and suggest a role for IL-21 in initial HIV-1 control *in vivo*.

## Abstract (web summary)

HIV-infected patients who maintain undetectable virus levels possess elevated plasma concentrations of IL-21. Here, Adoro *et al*. show that IL-21 inhibits early viral infection in humanized mice and suppresses HIV-1 replication *in vitro* by upregulating a microRNA via the regulatory protein STAT3.

## Graphical abstract

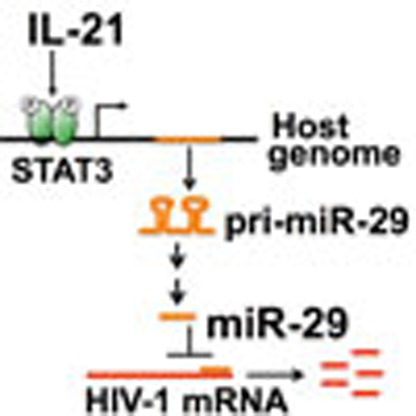

The first series of host and virus-induced events after HIV-1 exposure are critical for shaping the outcome of long-term HIV-1 infection and the development of AIDS^1^. Because of the small numbers of infected ‘founder’ CD4 T-cell populations implicated in initial HIV-1 transmission, the initial period following exposure has been suggested to present the most vulnerable stage to intervene against the virus^2^. As part of the host first line of defense against infection, cell-intrinsic factors that interfere with different stages of lentiviral replication represent potent mechanisms that can be harnessed to curtail initial infection before specific adaptive immune responses develop.

Interleukin-21 (IL-21), which is produced by activated CD4 T cells, belongs to the common gamma chain (γ_c_)-dependent family of cytokines^3^. While mouse models genetically deficient for this cytokine or its cognate receptor established its critical requirement in murine antiviral responses in chronic viral infections^4^,^5^,^6^, how IL-21 contributes to the initial immunity against major human viral infections like HIV-1 is less clear. The depletion of IL-21-producing CD4 T cells in progressive HIV-1 disease^7^,^8^,^9^ has raised the question whether IL-21 deficiency contributes to overall HIV-1 pathogenesis. In progressive untreated HIV-1 disease, higher frequencies of virus-specific IL-21-producing CD4 T cells were associated with viral control^7^,^8^,^9^ and HIV-1-infected ‘elite controllers (EC)’ who maintain undetectable viremia without antiretroviral therapy show elevated plasma levels of IL-21 and IL-21-producing T cells^7^,^8^,^10^. *Ex vivo* stimulation assays suggested that the mechanism of IL-21 activity involved its ability to promote perforin and granzyme expression in HIV-specific cytotoxic T cells^7^,^8^,^9^,^11^,^12^. However, as protective virus-specific cellular responses promoted by IL-21 develop several weeks after HIV-1 exposure^13^ this mechanism would not operate during the initial days after exposure.

Mature miRNA are 19–25 nucleotide duplexes generated from primary miRNA precursors (pri-miRNA) and are transcribed from genomic DNA sequences by RNA polymerase II^14^. Through splicing events catalysed by the RNase-III type enzymes Drosha and Dicer, pri-miRNA are processed into mature miRNA whose function is to destabilize target mRNA and suppress translation^15^. There is increasing evidence that cellular miRNAs play critical roles in HIV-1 pathogenesis including promoting viral infection, latency in resting CD4 T cells and mediating cell-intrinsic HIV-1 resistance^16^. While recent *in vitro* studies identified the miR-29 family as inhibitors of HIV-1 production and infectivity^17^,^18^, the significance of miR-29 activity on primary HIV-1 infection and the upstream signals that regulate miR-29 transcription in target CD4 T cells are not known.

Human lymphoid organ aggregate cultures (HLAC) have emerged as powerful *in vitro* systems to dissect early events during HIV-1 exposure in more physiological settings given the susceptibility of lymphoid CD4 T cells to HIV-1 infection without the need for mitogen stimulation that can potentially mask native virus–host cell dynamics^19^,^20^. Here we take advantage of the HLAC systems to investigate the role of IL-21 in initial HIV-1 resistance by CD4 T cells. We report that IL-21 suppresses initial HIV-1 infection in lymphoid CD4 T cells and this antiviral activity was rapid, independent of cytotoxic effector T cells, but requires induction of cell-intrinsic miR-29. Consistent with this antiviral activity, we find that exogenous IL-21 administration limits both the incidence and magnitude of primary HIV-1 infection *in vivo* in humanized mice.

## Results

### IL-21 directly suppresses HIV-1 infection in CD4 T cells

Given the critical role of IL-21 in viral immunity and its association with HIV-1 disease control^7^,^8^,^9^,^10^,^11^,^12^, we sought to investigate whether IL-21 contributed to the initial host response to HIV-1. Unlike CD4 T cells from peripheral blood, CD4 T cells in spleen or lymph node-derived HLAC do not require mitogenic stimulation for HIV infection and thus more closely mimic natural infection conditions^19^,^20^. We took advantage of this system to assess the effect of IL-21 on primary HIV-1 infections in HLACs prepared from freshly excised human splenic tissues (Supplementary Fig. 1a). HLACs were pretreated with IL-21 and infected with replication competent CCR5-tropic (R5-HIV–green fluorescent protein (GFP)) or CXCR4-tropic (X4-HIV–GFP) HIV-1_NL4-3_-encoding green fluorescent protein (GFP) to allow for direct quantification of infection by flow cytometry (Supplementary Fig. 1). HIV-1 infection was assessed by GFP expression, p24 proteins in culture supernatants and/or HIV-1 mRNA 72 h after infection, a time point preceding CD4 T-cell depletion in HLACs^19^.

**Figure 1:**
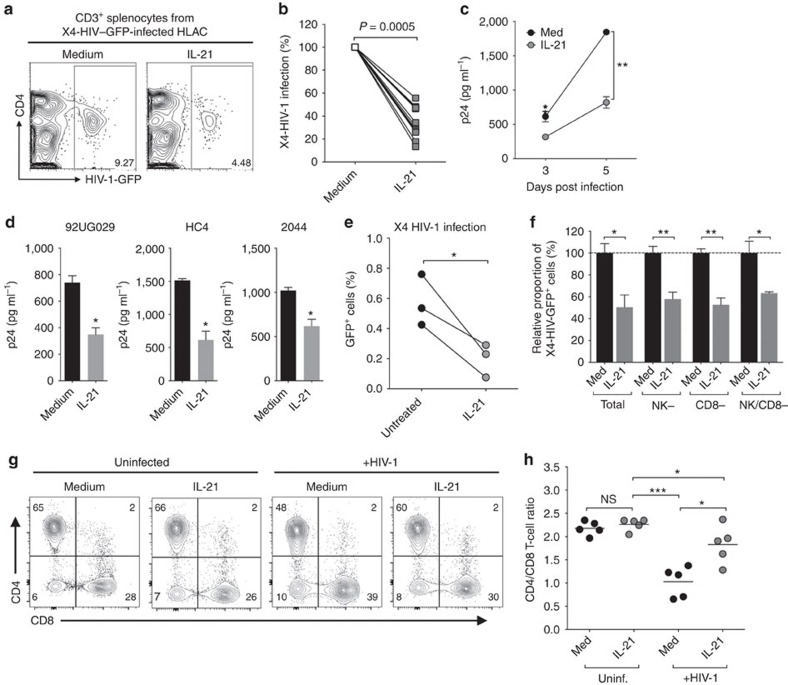
IL-21 suppresses initial HIV-1 infection in lymphoid CD4 T cells. (**a**) GFP versus CD4 expression on gated CD3^+^ cells in untreated (medium) or IL-21-treated HLACs 72 h post infection with GFP-tagged HIV-1_NL4-3_. (**b**) X4-HIV-1 infection (GFP^+^ cells gated as in **a**) in HLAC across multiple donors after 72 h. Infection in IL-21 treated cultures is expressed relative to untreated (medium, 100%). Each data point represents infection in HLAC prepared from individual donors (*n*=12) and compared by a paired Wilcoxon signed-rank test (*P*<0.0005). (**c**) HIV-1 p24 in HLAC supernatant at 3 and 5 days post infection. (**d**) HIV-1 p24 protein in HLAC supernatants infected with primary HIV-1 clades 72 h post infection. (**e**) Frequency of GFP^+^ cells in HLACs infected for 12 h with HIV-1 and washed extensively to remove viral supernatants before IL-21 treatment. Each data point represents a single donor. (**f**) Proportion of X4-HIV–GFP^+^ T cells in NK, NKT and/or CD8 T-cell-depleted HLAC determined as in **b**. Specific cell populations were depleted using commercially available anti-CD8 or anti-CD56 depleting microbeads as described in Methods. (**g**,**h**) Representative CD4 versus CD8 flow cytometry plot of CD3^+^ human splenocytes (**g**) and CD4/CD8 T-cell ratio (**h**) in uninfected (‘Uninf.’) or HIV-1-infected HLACs in the presence or absence (Medium, Med) of IL-21 6 days after HIV-1 inoculation. Each data point represents one donor. Data in **c**–**e** and **g** are means (± s.e.m.) of triplicate wells and are representative of three donors. *P<0.05; **P<0.005, ***P<0.0005; unpaired Students *t*-test.

Interestingly, we found that HIV-1 infection (as measured by GFP^+^CD3^+^) of CD4 T cells in IL-21-treated HLACs was significantly reduced compared with untreated cultures (Fig. 1a). Notably, compilation of X4-HIV-1 infection across multiple donors revealed marked suppression of HIV-1 infection by IL-21 (median suppression=68%, *n*=12; *P*<0.0005, Wilcoxon signed-rank test; Fig. 1b). The antiviral activity of IL-21 was independent of envelope tropism as IL-21 also suppressed R5-tropic HIV-1_NL4-3_ virus infection in HLACs (Supplementary Fig. 2a). Consistent with impaired infection, levels of HIV-1 p24 protein were significantly reduced in supernatants from IL-21-treated HLACs compared with untreated controls (Fig. 1c and Supplementary Fig. 2b), indicating that HIV-1 replication and overall viral production from infected cells were suppressed by IL-21.

We also tested the activity of IL-21 against infection with primary HIV-1 isolates. As shown in Fig. 1d, IL-21 also suppressed infection by primary clade A (92UG029) and clade B (HC4 and 2044) HIV-1-subtype-infected HLACs indicating a broad antiviral activity. IL-21 suppressed HIV-1 infection whether added before HIV-1 inoculation (Fig. 1a–d) or added to HLACs after HIV-1 inoculation and extensive washing to remove viral supernatants (Fig. 1e).

Since IL-21 can also promote virus-specific cytotoxicity through CD8 T cells and NK cells^12^,^21^, we asked whether its suppression of HIV-1 infection in HLACs was dependent on these cells. To this end, we depleted NK, NKT and/or CD8 T cells from HLACs before IL-21 treatment and virus inoculation (Supplementary Fig. 3). HIV-1 infection was equivalently suppressed by IL-21 even in the absence of NK, NKT and CD8 T cells (Fig. 1f), indicating that the observed antiviral activity of IL-21 did not depend on these cells but likely a direct effect on HIV-1 permissive CD4 T cells.

HLACs infected with HIV-1 show significant CD4 T-cell depletion around 5 days after infection^19^,^20^. To explore the significance of IL-21-mediated viral inhibition, we assessed CD4 T-cell depletion in HLACs in the presence and absence of IL-21 6 days post infection. Notably, we found that lower HIV-1 infection in IL-21-treated HLACs resulted in improved CD4/CD8 T-cell ratio (less CD4 depletion) in these cultures (Fig. 1g,h). Together, these findings suggest for the first time the ability of IL-21 to directly suppress HIV-1 infection in CD4 T cells. Furthermore, because CD4 T-cell depletion in HLACs is driven by cell death of abortively infected CD4 T cells, the data also suggest that IL-21 might broadly inhibit both productive and abortive viral infection by IL-21.

### IL-21 promotes antiviral miR-29 biogenesis

We next addressed the mechanism of IL-21-mediated inhibition. IL-21 inhibited both R5- and X4-tropic HIV-1 infection (Fig. 1 and Supplementary Fig. 2a), and did not alter surface expression of the HIV entry coreceptors CD4, CCR5 or CXCR4 on CD4 T cells (Supplementary Fig. 4a), suggesting that the antiviral mechanism of IL-21 likely involved post-entry events. Protein expression analysis revealed only a modest effect of IL-21 on the expression of classical HIV-1 restriction factors APOBEC3G, SAMHD1 and Tetherin (CD317)^22^ in splenic CD4 T cells (Supplementary Fig. 4a,b). The effect of IL-21 was also independent of cell proliferation, as cytokine treatment did not affect lymphoid CD4 T-cell proliferation status as determined by Ki-67 expression (Supplementary Fig. 4c). Nevertheless, the fact that IL-21 promoted such an early anti-HIV-1 response hinted that it likely modulated cell-intrinsic factors that directly antagonize HIV-1 replication in CD4 T cells.

Consequently, we wondered whether IL-21 regulated expression of the HIV-1-restrictive miR-29 family^17^,^18^, a cluster of highly conserved and co-regulated miRNA that includes miR-29b1/29a and miR-29b2/29c on human chromosome 7 and 1, respectively^23^. Consistent with its reported ability to inhibit HIV-1 production and infectivity in HEK293T and HeLa cell lines^17^,^18^, we found that overexpression of miR-29b in human Jurkat T-cell lines using lentiviral vectors significantly inhibited infection of HIV-1_NL4-3_-luciferase as measured by enzymatic luciferase activity^24^ (Supplementary Fig. 5a). Conversely, using the human CD4 T-cell line CEM-GXR25 that expresses GFP driven by HIV-1 LTR in a Tat-dependent manner^25^, we confirmed that constitutive inhibition of endogenous miR-29 by lentivirus-encoded antisense ‘miR-29-ZIP’ enhanced infection with CXCR4-tropic HIV-1_NL4-3_ (Supplementary Fig. 5b). These results demonstrate that endogenous miR-29 critically regulates the extent of HIV-1 infection of CD4 T cells and suggest that miR-29 likely inhibits early HIV-1 replication steps.

**Figure 2:**
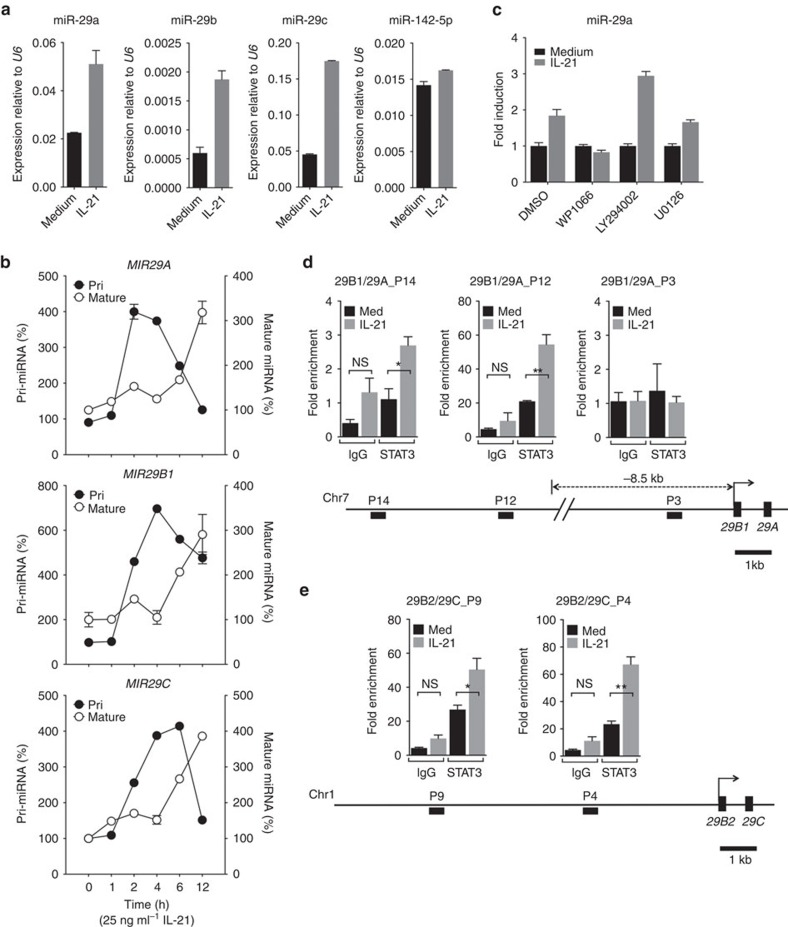
IL-21 induces antiviral miR-29 through STAT3. (**a**) Stem-loop-specific PCR quantification (average±s.d. of duplicate wells) of mature miR-29a, miR-29b, miR-29c and miR-142-5p in purified total human splenic CD4 T cells from untreated (medium) or IL-21-treated HLACs after 12 h. (**b**) Kinetics of expression (average±s.d. of duplicate wells) of pri-miR-29 (normalized to *ACTB*) and mature miR-29 (normalized to *U6*) species in purified CD4 T cells isolated from IL-21-treated splenocytes. Values are percentage induction expressed relative to time 0 (=100%). (**c**) Effect of pharmacological inhibitors against STAT3 (WP1066), PI3-kinase (LY294002) and MAP/Erk kinase (U0126) on IL-21-mediated miR-29 gene induction. (**d**,**e**) ChIP assay for STAT3 binding to chromosome 7 *MIR29B1/MIR29A* (**d**) and chromosome 1 *MIR29B2/MIR29C* (**e**) genes in primary human splenic CD4 T cells. Fold enrichment (average±s.e.m. of triplicate wells) were determined relative to position P3, which showed no STAT3 enrichment by PCR (**d**). Binding of STAT3 to the IL-21/STAT3 target gene *SOCS3* was used as a positive control P3 in *MIR29B1/MIR29A* gene. Black bars (∼500 bp) indicate regions containing primers with detectable amplification of ChIP DNA. ChIP was performed on purified CD4 T cells pooled from three donors to obtain sufficient DNA. Data are representative of two (**b**,**c)** and five donors (**a**). *P<0.05; **P<0.005; NS, not significant; unpaired Students *t*-test.

Using miRNA species-specific quantitative PCR, we detected markedly upregulated expression of all mature miR-29 species in CD4 T cells in IL-21-treated splenic CD4 T cells from HIV-1-negative donors (Fig. 2a). By contrast, TCR/CD28 activation of CD4 T cells, which promotes HIV infection^1^, downregulated miR-29 (Supplementary Fig. 6a). IL-21 did not modulate expression of miR-142-5p, which is expressed in haematopoietic cells^26^ (Fig. 2a), indicating that it acted selectively on the miR-29 locus and not on miRNAs generally.

Because productive HIV-1 infection occurs in activated and/or memory CD4 T cells^1^,^19^,^27^, we next asked if IL-21 specifically promoted miR-29 expression in these permissive CD4 T-cell populations. While splenic CD4 T cells activated with plate-bound anti-CD3 and anti-CD28 antibodies downregulated miR-29, IL-21 treatment still promoted miR-29 expression in these cells (Supplementary Fig. 6a). We similarly assessed miR-29 expression in total, naïve and memory CD4 T cells, which include highly HIV-1-permissive CD4 T cells^27^,^28^. Even though memory CD4 T cells contained lower levels of miR-29 compared with naïve T cells, they significantly upregulated miR-29 in response to IL-21 (Supplementary Fig. 6b). These results are significant as they demonstrate induction of miR-29 by IL-21 in HIV-1 permissive CD4 T cells that account for productive HIV-1 infection.

To elucidate how IL-21 promoted miR-29 expression, we assessed whether it regulated early steps in miR-29 biogenesis. Quantification of pri-miR-29 transcripts revealed that IL-21 promoted the first step in the miR-29 biogenesis pathway as stimulation with IL-21 increased expression of pri-miR-29 transcripts peaking at about 4 h (Fig. 2b and Supplementary Fig. 7a). Pri-miR-29 induction preceded the accumulation of mature miR-29 species, which peaked at about 12 h (Fig. 2b). These results identify IL-21 as a regulator of miR-29 biogenesis and suggest that IL-21-mediated inhibition of primary HIV-1 infection was effected through miR-29.

### Induction of miR-29 by IL-21 is STAT3 dependent

On binding to its heterodimeric receptor composed of IL-21Rα and γ_c_-chain, IL-21 activates STAT3, PI3-kinase as well as the MAPK/Erk pathways^3^. Thus, to elucidate how IL-21 regulated miR-29 expression, we blocked individual signalling pathways downstream of IL-21R in IL-21-treated splenic CD4 T cells. Pharmacological inhibition of STAT3 with the inhibitor WP1066 but not other downstream IL-21R signalling pathways abrogated IL-21-mediated induction of miR-29, implicating STAT3 in *MIR29* gene induction (Fig. 2c and Supplementary Fig. 7b).

To determine specifically whether STAT3 regulates miR-29 transcription, we performed chromatin immunoprecipitation (ChIP) assay with anti-STAT3 antibody on untreated or IL-21-treated primary human splenic CD4 T cells (Figs 2d,e). As a positive control, we detected significant STAT3 binding upstream of exon 1 of *SOCS3* (Supplementary Fig. 7c), an IL-21/STAT3 target gene^29^. Quantitative PCR analysis with primers across an ∼15 kb upstream of *MIR29* showed significantly enriched STAT3 binding to two putative regulatory regions upstream of *MIR29B1/29A* after IL-21 treatment (Fig. 2d). STAT3 binding was also enriched at two regions upstream of *MIR29B2/29C* in IL-21-treated splenic CD4 T cells (Fig. 2e). Together, these results strongly suggest that the IL-21-activated STAT3 transcription factor contributes to the induction of miR-29 genes in CD4 T cells.

### IL-21-mediated HIV-1 suppression requires miR-29

**Figure 3:**
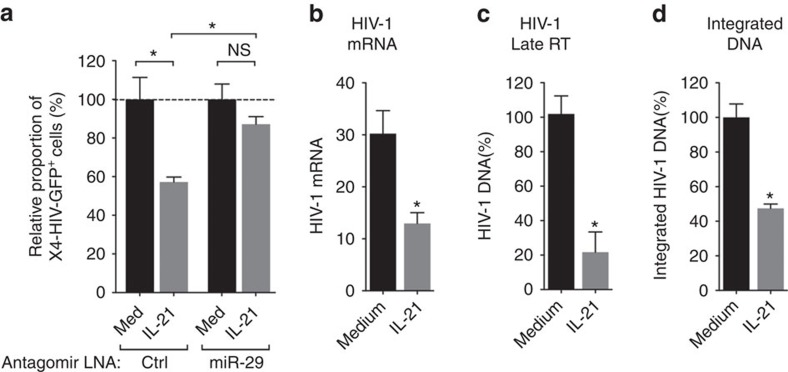
Mir-29 is required for IL-21-mediated viral suppression. (**a**) Proportion of HIV–GFP^+^ cells (gated as in Fig. 1a) in untreated (‘Med’) or IL-21-treated HLAC reconstituted with control (Ctrl) or miR-29 antagomir-LNA-treated CD4 T cells 72 h after infection with X4-HIV-1_NL4-3_. (**b**) HIV-1 mRNA quantification (normalized to *ACTB*) in purified HLAC CD4 T cells 72 h post infection. (**c**,**d**) Cell-associated HIV-1 DNA. Late reverse transcripts (RT) HIV-1 DNA (**c**) and integrated HIV-1 DNA (**d**) were determined in purified CD4 T cells from HLACs after 72 h of infection with HIV-1_NL4-3._ Data are average (±s.e.m.) of two donors and normalized to untreated (medium, 100%) control wells. *P<0.05; NS, not significant; Students *t*-test.

To determine whether IL-21-mediated suppression of HIV-1 infection required miR-29, purified splenic CD4 T cells were nucleofected with synthetic miR-29 ‘antagomir’ locked nucleic acids (LNA). Antagomirs are complementary to and inhibit miRNA activity by sequestering them from their target mRNA^30^. Intracellular delivery of miR-29 antagomir LNA and inhibition of miR-29 activity in CD4 T cells was confirmed by the enhanced expression of *TBX21* and *IFNG*, two miR-29-repressed genes^31^ (Supplementary Fig. 8). Nucleofected CD4 T cells were then admixed with autologous CD4-depleted HLAC, infected with HIV-1_NL4-3_ and evaluated for HIV-1 infection by GFP expression (Supplementary Fig. 1). Compared with control-LNA antagomirs, mir-29-LNA antagomirs significantly abrogated the ability of IL-21 to inhibit HIV-1 infection in CD4 T cells (Fig. 3a), indicating that the antiviral activity of IL-21 was at least in part mediated by miR-29. Of note, in contrast to interferon-α (IFNα) that suppressed HIV-1 replication through classical cell-intrinsic restriction factors^22^, the antiviral activity of IL-21 coincided with its ability to induce miR-29 compared with other Th1 and Th17 effector cytokines (Supplementary Fig. 9a). Consistent with their ability to activate STAT3, IL-6 and IL-10 also induced miR-29 and suppressed HIV-1 infection in HLACs (Supplementary Fig. 9b). However, within the same donor, IL-21 was a more potent suppressor of HIV-1 compared with IL-10 and IL-6.

To further explore the mechanism of the antiviral activity of IL-21, we quantified viral genes at different stages of the HIV-1 life cycle in purified CD4 T cells from HLACS. Importantly, RNA samples were DNase I treated before reverse transcriptase reaction to remove contaminating HIV-1 DNA. Quantitative PCR analysis revealed significantly reduced HIV-1 mRNA expression in purified CD4 T cells from infected HLACs treated with IL-21 compared with medium alone (Fig. 3b). We also quantified HIV-1 DNA in total DNA templates from infected cells. Similarly, fewer HIV-1 late reverse transcripts (RT) and integrated HIV-1 DNA were detected in IL-21-treated samples (Figs 3c,d), suggesting that the IL-21–miR-29 axis interfered with early HIV-1 replication steps. Collectively, these findings demonstrate a novel and rapid miR-29-mediated antiviral activity of IL-21 that acts through STAT3 in target CD4 T cells to limit initial HIV-1 infection.

### IL-21 reverses HIV-1-induced miR-29 downregulation

**Figure 4:**
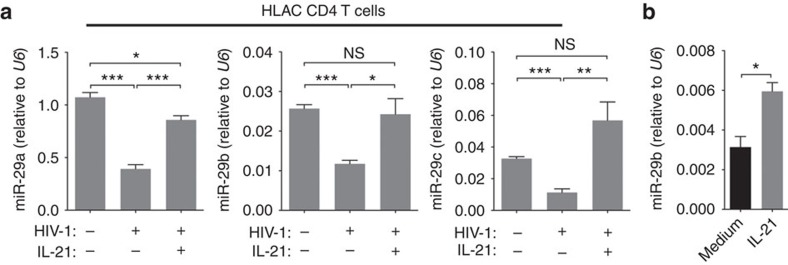
IL-21 reverses HIV-1-induced miR-29 downregulation. (**a**) Expression of miR-29 species (average±s.d. of duplicate wells) in CD4 T cells in untreated or IL-21-treated HLACs 72 h post infection with HIV-1_NL4-3_. MicroRNA-29 was quantified in total CD4 T cells purified from the respective culture conditions 72 h after HIV-1 infection. Data are representative of three donors. (**b**) MicroRNA-29b expression (average±s.d. of duplicate wells) in CD4 T cells from a viremic HIV-1-infected spleen. MicroRNA-29 levels in purified CD4 T cells after 16 h of treatment with IL-21. *P<0.05; **P<0.005; ***P<0.0001; NS, not significant; Students *t*-test.

Interestingly, we found that CD4 T cells from HIV-1-infected HLACs showed a marked downregulation of all miR-29 species (Fig. 4a), suggesting that virus-induced mechanisms downregulated miR-29 transcription. Because IL-21 impaired HIV-1 infection when administered before or after viral exposure, we asked whether it can reverse HIV-1-associated downregulation of miR-29. To address this question, we compared miR-29 expression in purified CD4 T cells isolated from untreated or IL-21-treated HLACs that were equally infected with HIV-1. As shown in Fig. 4a, treatment with IL-21 completely restored expression of all miR-29 species. These results indicate that IL-21 is able to overcome miR-29 downregulation during HIV-1 infection and that HIV-1 infection did not impair IL-21R signalling in CD4 T cells. Indeed, we confirmed that splenic CD4 T cells from an HIV-1-infected subject significantly upregulated miR-29 in response to IL-21 treatment (Fig. 4b). Importantly, the ability of IL-21 to overcome HIV-1-induced miR-29 downregulation was consistent with its ability to also suppress HIV-1 infection when added to HLACs after CD4 T cells have been infected with HIV-1 (Fig. 1e).

### MicroRNA-29 is associated with control of HIV-1 disease

**Figure 5:**
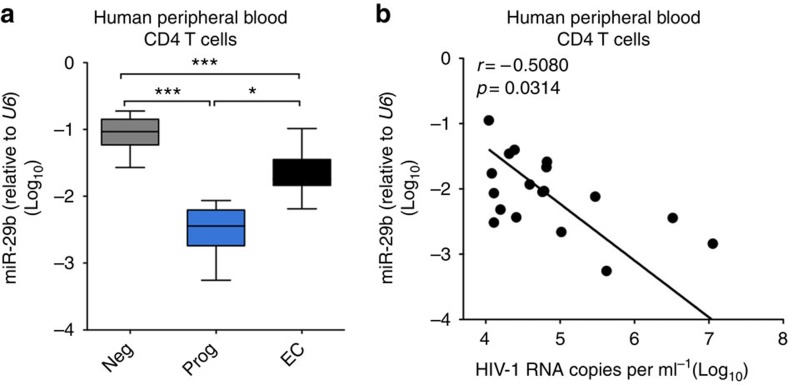
Relationship between miR-29 expression and HIV-1 disease in humans. (**a**) Expression of miR-29b in HIV-negative (Neg, *n*=10), HIV-1-infected untreated progressors (Prog, *n*=19) and elite controllers (EC, *n*=10). Data are compared with the unpaired Student’s *t*-test. (**b**) Correlation between miR-29b and plasma HIV-1 titer in untreated HIV-1-infected progressors. Each data point represents an individual and Spearman’s correlation coefficient (*r*) and *P* values are shown.

Our preceding results strongly supported a significant contribution of an IL-21–miR-29 axis in HIV-1 control prompting us to next determine the relationship between miR-29 and HIV-1 infection in human subjects. We quantified miR-29 expression in peripheral blood CD4 T cells from uninfected, treatment naïve HIV-infected progressors (Prog) and EC who suppress HIV in the absence of antiretroviral drugs^32^. Similar to CD4 T cells from HIV-1-infected HLACs (Fig. 4a), we detected marked downregulation of miR-29b expression in CD4 T cells from untreated HIV-infected Progs compared with uninfected and ECs subjects (Fig. 5a). Notably, among untreated HIV-infected Progs, miR-29b expression inversely correlated (Spearman correlation coefficient (*r*)=−0.5080; *P*=0.0314) with plasma HIV titres (Fig. 5b). Together, these results from human subjects are consistent with a protective role for miR-29 in HIV-1 control and suggest that downregulated miR-29 expression could represent a signature of progressive HIV-1 disease.

### Exogenous IL-21 limits early HIV-1 infection *in vivo*

**Figure 6:**
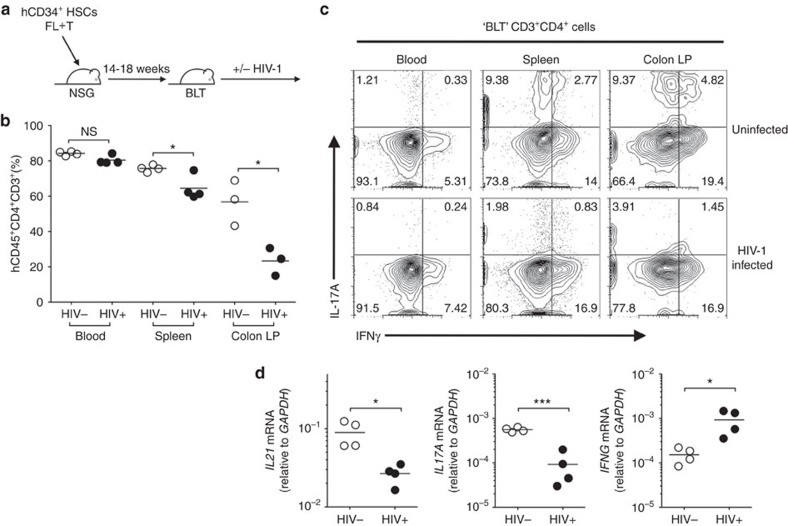
Characterization of acute HIV-1 infection in ‘BLT’ humanized mice. (**a**) Generation and infection of BLT humanized mice schema. (**b**) Proportion of human CD4 T cells in peripheral blood, spleen and colon lamina propria (LP). (**c**) Flow cytometry analysis of intracellular IL-17A and IFNγ protein expression in CD3^+^CD4^+^ T cells from the indicated tissues from uninfected (top) or HIV-1-infected (bottom) humanized mice after 6 weeks. Single cell suspensions from the indicated tissues were stimulated with PMA and ionomycin for 5 h. Numbers represent proportion of cells within each gate. (**d**) Quantification of *IL21*, *IL17A* and *IFNG* mRNA (normalized to *GAPDH*) in splenic CD4 T cells from BLT mice 6 weeks post HIV-1 infection (four mice per group). *P<0.05; ***P<0.0001; unpaired Students *t*-test.

Finally, we sought to investigate the effect of exogenous IL-21 treatment on primary HIV-1 infection *in vivo*. Humanized mice show robust *in vivo* HIV-1 infection, recapitulate key events of HIV-1 pathogenesis^33^,^34^ and represent a tractable animal model to interrogate the early antiviral activity of IL-21 *in vivo*. However, whether humanized mice show cytokine dynamics comparable to acute HIV-1 infection in humans is not known. Thus, we sought to first define the nature of acute HIV-1 infection in humanized mice. We generated ‘BLT’ (bone marrow, foetal liver and thymus) humanized mice by engrafting NOD/SCID/γ_c_-deficient mice with human foetal liver, thymus and autologous bone marrow CD34^+^ haematopoietic stem cells^33^ (Fig. 6a). Fully reconstituted humanized BLT mice were infected with 10,000 TCID50 of CCR5-tropic HIV-1_JR-CSF_ clone and assessed for HIV-1 infection 4–6 weeks post infection. While acute HIV-1 infection presented with marginal CD4 T-cell depletion in blood and spleen of humanized mice, CD4 T cells were severely depleted in gut lamina propria (Fig. 6b). Because acute lentiviral infections modulate IL-17A and IL-21-producing CD4 cell populations^8^,^35^, we evaluated IL-17A, IL-21 and IFN-γ cytokine expression by intracellular flow cytometry and quantitative PCR in acutely infected BLT mice. Importantly, we found that acute HIV-1 infection in BLT mice resembled human HIV-1 infection, since there was loss of IL-21-8,36 and IL-17A-producing^35^,^37^ CD4 T cells reflected in the downregulation of *IL21* and *IL17A* mRNAs and proteins from CD4 T cells (Fig. 6c,d). Thus, we considered the humanized BLT mice a useful model to interrogate the *in vivo* role of IL-21 in HIV-1 infection.

**Figure 7:**
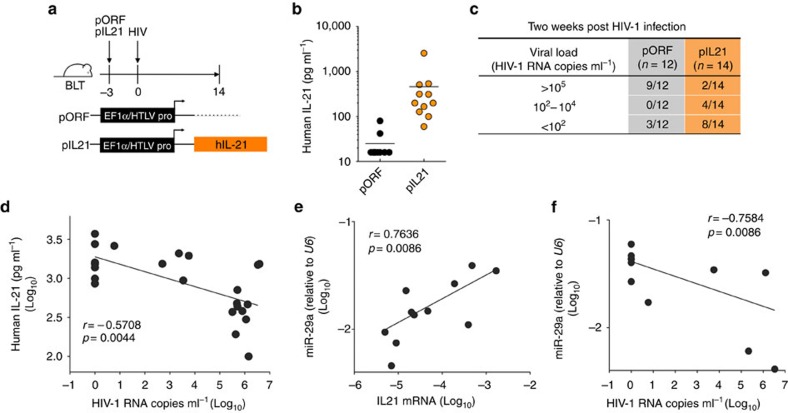
Exogenous IL-21 limits early HIV-1 infection in BLT humanized mice. (**a**) Experimental design: pORF, control plasmid; pIL21, human IL-21-encoding plasmid. (**b**) Concentration of human IL-21 in plasma of humanized mice 3 days after hydrodynamic injection. (**c**) Range of plasma HIV-1 RNA copies 2 weeks post infection. (**d**) Correlation between plasma IL-21 and HIV-1 titer in pooled pIL21-treated BLT humanized mice 2 weeks post infection. (**e**,**f**) Correlation between miR-29 and *IL21* mRNA (**e**) or plasma HIV-1 titer (**f**) in humanized mice 2 weeks post infection. Each data point represents a humanized mouse; Spearman’s correlation coefficient (*r*) and *P* values are indicated. Note that *x*- and *y*-axis (**d**–**f**) are log_10_ scales.

We set-up two groups of BLT humanized mice that were comparably reconstituted with human immune cells (human CD45^+^; Supplementary Fig. 10a). Using hydrodynamic injection, one group of mice was injected with control plasmid (pORF) and the other group was injected with human IL-21-encoding plasmid (pIL21) to induce systemic IL-21 expression (Fig. 7a). Compared with pORF-treated animals, pIL21-treated mice had augmented plasma IL-21 levels as early as 72 h after injection (Fig. 7b). Of note, BLT mice injected with pORF and pIL21 plasmids by hydrodynamic injection contained comparable proportions of peripheral human CD3^+^ cells, human CD4^+^ and CD8^+^ T cells (Supplementary Fig. 10b,c), indicating that exogenous IL-21 did not modify human immune cell and overall T-cell reconstitution in these animals (Supplementary Fig. 10).

Seventy-two hours after hydrodynamic injection, both pORF and pIL21-treated humanized mice were infected with 10,000 TCID50 of CCR5-tropic HIV-1_JR-CSF_ clone and assayed for plasma viral load 2 weeks after infection, a time point preceding detectable virus-specific cellular and antibody responses in this model^33^. While 75% (9 out of 12) of control pORF-treated animals had >10^5^ HIV-1 RNA copies per ml plasma, only 14% (2 out of 14) of pIL21-treated mice had >10^5^ viral titres (Fig. 7c). In fact, most pIL21-treated animals presented with plasma HIV-1 titer <10^4^ HIV-1 RNA copies per ml, and 57% (8/14) showed HIV-1 titres <10^2^ HIV-1 RNA copies per ml (Fig. 7c). Notably, HIV-1 titres inversely correlated with plasma IL-21 levels (Fig. 7d), supporting a protective role for IL-21 against very early HIV-1 infection.

To understand the contribution of miR-29 to IL-21-mediated suppression in early HIV-1 protection in BLT humanized mice, we evaluated the expression of miR-29 and IL-21 in splenic CD4 T cells from IL-21-treated and HIV-1-infected animals. Not only did *IL21* mRNA levels correlate with miR-29 in splenic CD4 T cells (Fig. 7e), we observed a significant inverse correlation between miR-29 expression and plasma HIV-1 titres (Fig. 7f). Taken together, these results implicate an IL-21–miR-29 axis in direct HIV-1 suppression in CD4 T cells and suggest that pretreatment with IL-21 can limit the magnitude of the initial HIV-1 infection *in vivo*.

## Discussion

In this study, we report a novel antiviral activity of IL-21 that is mediated by miR-29 and results in suppressed HIV-1 infection in primary lymphoid CD4 T cells. Interestingly, we found that humanized mice treated with exogenous IL-21 were largely protected from early HIV-1 infection and reduced viral load in these animals correlated with higher miR-29 expression in splenic CD4 T cells. Unlike its reported role in promoting virus-specific cytotoxic responses in HIV-1 disease^7^,^8^,^9^,^10^,^11^,^12^, the activity of IL-21 in suppressing initial HIV-1 infection in CD4 T cells was independent of cytotoxic CD8, NK and NK T cells but dependent on cell-intrinsic miR-29 species, which suppress HIV-1 production and infectivity^17^,^18^. Mechanistically, miR-29 binds to seed regions in the 3′-untranslated region of HIV-1 mRNA and targets it to cellular P bodies resulting in viral mRNA degradation and suppression of translation^18^. MicroRNA-29 also downregulates HIV *nef* transcripts and nef protein expression which, as nef is essential for optimal HIV-1 production, compromises overall HIV-1 production^17^. Consistent with these modes of action, we indeed observed that CD4 T cells from IL-21-treated HLACs had lower HIV-1 mRNA transcripts and HIV-1 DNA.

IL-21 is produced by activated T cells including Th17 cells, T follicular helper (T_fh_) cells, NK T cells and, in some instances, CD8 T cells^3^. Our present results implicate a significant role for cellular sources of IL-21 in initial HIV-1 control, a role that precedes and complements its function in promoting virus-specific T-cell responses^7^,^12^. Studies in HIV and SIV models reveal a complex dynamic of IL-21-producing T cells over the course of infection with evidence that IL-21-producing CD4 T cells^8^,^36^, including Th17 lineage cells^35^,^37^, are depleted in acute lentiviral diseases. Given the ability of IL-21 to promote HIV-1 resistance in CD4 T cells through miR-29, it is likely that depletion of IL-21-producing T cells compromises this line of defense against HIV-1, which would suggest that higher pre-infection levels of IL-21-producing cells would indicate better HIV-1 prognosis. Indeed, it was recently reported that higher pre-existing levels of Th17 cells (an IL-21-producing CD4 T-cell lineage^38^,^39^) were associated with lower viral set-point in SIV-infected rhesus macaques^40^. Future similar prospective studies in patients detailing how pre-infection levels of IL-21-producing cells correlate with the outcome of HIV-1 exposure would be valuable in fully understanding the prognostic significance of IL-21 during HIV-1 disease.

Infection with HIV-1 induces profound changes in the miRNA landscape in infected CD4 T cells and these changes include mechanisms that suppress antiviral miRNAs while facilitating viral replication^41^,^42^,^43^,^44^. Indeed, as we report here, severely reduced miR-29 expression is a hallmark of progressive HIV-1 disease^43^,^45^. That miR-29 is downregulated within 72 h after HIV-1 infection in lymphoid CD4 T cells suggests that it is likely mediated by virus-induced extrinsic factors (and not due to direct infection by HIV-1), given the very small fraction of HIV-1 susceptible CD4 T cells *in vivo*^1^,^2^. Interestingly, coincident with their higher levels of IL-21 expression^7^,^8^, ECs maintained higher miR-29 expression compared with progressive infection. Evidence that not all ECs express protective human leucocyte antigen (HLA) class I alleles^32^ and that expression of classical HIV-1 restriction factors are indistinguishable between Progs and ECs^46^ suggests that an IL-21–miR-29 axis might represent a non-classical HIV-1 restriction mechanism in these individuals.

The molecular details of miR-29 gene regulation are not well defined but appear to be disease and cell-type specific and likely involves proximal and long-range distal acting *cis* elements^23^. The mechanism of miR-29 downregulation in activated and memory CD4 T cells might involve nuclear factor kappa-light-chain-enhancer of activated B cells (NF-κB) as stimulation with anti-TCR/CD28 antibodies (which downregulated miR-29) activates NF-κB, a repressor of miR-29 gene expression in myoblasts^47^. Our study is the first to describe an IL-21/STAT3 axis as a regulator of miR-29 transcription. Abrogation of IL-21-mediated miR-29 induction on STAT3 blockade coupled with enriched STAT3 binding to putative regulatory sites within *MIR29* genes support STAT3 as a positive regulator of miR-29 expression in CD4 T cells during HIV-1 infection. Notably, the STAT3-binding regions we identified overlapped with highly conserved sequences and predicted DNase I hypersensitivity sites in *MIR29* genes^47^. In further support of its STAT3 dependency, the STAT3-activating cytokines IL-6 and IL-10 also promoted miR-29 expression. However, because the proinflammatory activity of IL-6 is associated with HIV-1 disease progression^48^ and IL-10 is immunosuppressive towards immune cells and promotes CD4 T cell dysfunction in HIV-1 disease^49^, whether—and when during infection—either cytokine will contribute to overall HIV-1 control *in vivo* remains to be investigated. Nevertheless, these observations together suggest that STAT3-mediated signalling is an important aspect of the initial anti-HIV-1 response in CD4 T cells.

HLACs provide more physiologically relevant HIV-1 infection conditions since, unlike peripheral blood CD4 T cells lymphoid CD4 T cells are susceptible to HIV-1 infection without mitogenic stimulation. This is presumably because of the cellular composition of lymphoid organs and dispersed lymphoid organ cultures, which include innate and stromal cell populations that provide a cell–cell interaction network, which facilitates virus binding and entry into lymphoid CD4 T cells^19^,^20^. HLACs were particularly invaluable to our study because mitogen-activated CD4 T cells not only downregulated miR-29 but typically also produce cytokines including IL-2 and IL-15, which promote lentivirus entry^50^, potentially obscuring the antiviral effect of IL-21. Indeed, IL-21 had no effect on HIV-1 infection in peripheral blood mononuclear cells (PBMCs) activated with the mitogen phytohaemagglutinin^21^, but it substantially reduced HIV-1 infection in HLAC CD4 T cells as reported here.

IL-21 inhibited HIV-1 production through suppression of viral mRNA transcription and protein expression and was equally effective across different HIV-1 clades. Of note, we found that pre-infection treatment with IL-21 significantly reduced the extent and incidence of HIV-1 infection in humanized mice even though a previous study reported only a marginal effect of IL-21 on viral titres in SIV-infected rhesus macaques^51^. Because IL-21 was administered 2 weeks after SIV infection at peak viremia in that study^51^, it is possible that already established lentiviral infections escape protective mechanisms induced by IL-21 (including miR-29) rendering this cytokine ineffective at curbing virus dissemination.

Compared with secondary lymphoid organs, mucosal tissues of the gut and reproductive tract, key sites of HIV-1 entry, replication and dissemination^2^ are enriched in unique populations of immune cells that are in constant cross-talk with resident commensal microbial communities^52^. Among these cells are the Th17 CD4 T-cell subsets, which secrete significant amounts of several cytokines including IL-17A, IL-21, IL-22 and IL-26 in the steady-state and in response to TCR activation, commensal antigens or cytokines like IL-23 produced by dendritic cells^38^,^52^,^53^,^54^. The eclipse phase of HIV-1 infection, which comprises the first 7 days after exposure and precedes significant CD4 T-cell depletion, has been proposed as a vulnerable stage for effective antiviral strategies given the small numbers of infected ‘founder’ CD4 T cells at the site of viral entry and the limited degree of systemic infection at this time^2^. Thus, we speculate that local concentrations of IL-21 produced by Th17 and other CD4 T cells in mucosal tissues would contribute to limiting the magnitude of HIV-1 replication and initial dissemination during this period. However, future work in experimental animal models that allow genetic ablation or depletion of IL-21/STAT3 before HIV-1 infection will be required to more precisely define the *in vivo* activity of IL-21 at early stages of lentiviral infections. In uncovering an antiviral IL-21–miR-29 axis that impairs early HIV-1 infection, our present study suggests that endogenous IL-21 and strategies that exogenously augment IL-21 or induce pre-existing cellular sources of IL-21 would be beneficial in not only promoting adaptive antiviral immunity but also contribute to limiting the magnitude of the initial HIV-1 infection.

## Methods

### Human subjects and ethical statement

HIV-negative and HIV-infected subjects were recruited from outpatient clinics at the Massachusetts General Hospital (MGH) and affiliated Boston area hospitals according to protocols approved by the Institutional Review Board of MGH and Weill Cornell Medical College. All subjects gave written informed consent prior to enrolment in the study. HIV-infected samples were defined by the following characteristics: untreated Progs (viral load, 11,000–11.3 × 10^6^ HIV-1 RNA copies per ml plasma; 361–1,193 CD4^+^ T cells per μl) and ECs (<50 HIV-1 RNA copies per ml plasma; 400–1,800 CD4^+^ T cells per μl). Spleen or lymph node were obtained as discarded tissues from HIV-negative patients undergoing routine surgical procedures at MGH and used fresh for all assays.

### Virus preparation and quantification

The proviral plasmid pBR-NL43-IRES–EGFP-*nef*^*+*^ (pIeG-nef^*+*^)^55^ for CXCR4-tropic ‘X4-HIV–GFP’ viruses was obtained from the NIH AIDS Research & Reference Reagent Program (ARRRP, Catalogue #11349). The ‘R5-HIV–GFP’ virus was constructed by replacing the V3 loop of *env* with the corresponding sequence from the R5 clone BaL and confer R5-tropism^56^. R5- or X4-HIV–GFP were produced by transfecting HEK293T cells with the proviral plasmids as previously described^57^. Viral supernatants were harvested after 48 h, clarified through a 0.22 μm filter and stored at −80 ^o^C until use. Viral stocks of the HIV-1_JR-CSF_ clone were produced through transfection of human embryonic kidney (HEK) 293T cells using MAGI.CCR5 cells as described previously^58^. NL4-3-luciferase HIV-1 (ref. 24) and primary HIV-1 clade A and B viruses were kindly provided by John P Moore and Thomas Ketas (Weill Cornell Medical College).

### Human lymphoid aggregate cultures

HIV-1 and HIV-2 negative human spleens were obtained from patients undergoing routine surgical procedures at MGH, processed into single cell suspensions and used to set-up HLACs within 24 h after harvest as previously described^19^ (Supplementary Fig. 1). Specifically, dispersed cell suspension were resuspended and cultured in RPMI medium supplemented with 2 mM L-glutamine, 1 mM sodium pyruvate, 10 mM HEPES, 1 × MEM nonessential amino acids, 50 IU ml^−1^ penicillin, 50 μg ml^−1^ streptomycin (Corning Cellgro) and 15% foetal bovine serum. For infection, 0.5–2 × 10^6^ cells were pretreated with the indicated cytokines at a final concentration of 25 ng ml^−1^ in v-bottom 96 well plates for 16–18 h. Subsequently, viral supernatants were added to wells and the plates were centrifuged at 1,200*g* for 2 h at room temperature and cultured for 72 h. Infection of splenocytes with R5-HIV–GFP was only possible in the presence of 8 μg ml^−1^ polybrene. We assessed infection after 72 h because of the minimal CD4 depletion at this time point^19^. HIV-1 infection was quantified by GFP expression by flow cytometry or by p24 protein expression in culture supernatants determined by the Allianz p24 ELISA kit (Perkin Elmer).

Following previously published approaches to deplete NK and CD8 T cells^59^,^60^, where indicated CD8^+^, CD56^+^ NKT and NK cells were depleted with human CD8 microbeads (Miltenyi Biotec, #130-045-201) and CD56 microbeads (Miltenyi Biotec, #130-050-401) according to the manufacturer’s protocol. Briefly, single cell splenocyte suspensions were resuspended in phosphate buffered saline (PBS) containing 0.5% bovine serum albumin (BSA) and 2 mM EDTA (PBS–BSA–EDTA). Cells were then incubated for 15 min at 4 ^o^C with either anti-CD8 microbeads, anti-CD56 microbeads or both anti-CD8 plus anti-CD56 at a ratio of 20 μl CD8 microbeads per 10^7^ total cells. After incubation, cells were washed and resuspended in 500 μl of PBS–BSA–EDTA buffer and applied to pre-equilibrated LS magnetic columns (Miltenyi Biotec, #130-042-401) placed in the magnetic field of MACS separator. Columns were washed and eluted with PBS–BSA–EDTA buffer and the eluted fraction containing the CD8- and CD56-depleted splenocytes were used for HLACs.

For microRNA depletion, total splenic CD4 T cells were purified using the untouched human CD4 T cell isolation kit (Miltenyi Biotec, #130-096-533) and then nucleofected using the unstimulated human T-cell protocol (VPA-1002; programme U-014; Nucleofector II) with 30 pmol of pooled antagomir LNA against miR-29 (Exiqon #4101448-101, #4100754-101) or control antagomir (Exiqon #199006-101), rested for 4–6 h and re-combined with the CD4-depleted (Miltenyi Biotec, #130-045-101) splenic HLAC fraction. HLACs where then treated with cytokine at a final concentration of 25 ng ml^−1^ and infected with HIV-1 (Supplementary Fig. 1). The following cytokines were used: recombinant murine IL-21, human (h) IFNγ; recombinant hIL-17A, hIL-23, hIFNγ and CCL20 (Peprotech); hIFNα (PBL Biomedical Laboratories). For microRNA quantification, total splenocytes were treated with the indicated cytokines overnight (12–16 h) except otherwise indicated after which total CD4 T cells were purified for total RNA isolation. In some cases, cultures were treated with 10 μM final of IL-21R signalling inhibitors (EMD Millipore) LY294002 (anti-PI3-Kinase), WP1066 (anti-STAT3) and U0126 (anti-MEK).

### Generation of BLT humanized mice

BLT (bone marrow/liver/thymus) humanized mice were generated as previously described^33^. Briefly, NOD/SCID/IL-2Rγ^−/−^ mice at 4 to 6 weeks of age were conditioned with sub-lethal (2 Gy) whole-body irradiation. They were anesthetized the same day, and ∼1 mm^3^ fragments of human foetal thymus and liver (17 to 19 weeks of gestational age; Advanced Bioscience Resources) were bilaterally implanted under the kidney capsules. Remaining foetal liver tissue was used to isolate CD34^+^ cells with anti-CD34 microbeads (Miltenyi Biotech), which then were injected i.v. (1–5 × 10^5^ cells per mouse) within 6 h. Peripheral blood percentages of human T cells reached a plateau at their maximal values between 14 and 18 weeks after reconstitution. All reconstituted mice used for experiments were included if >40% of cells in the lymphocyte gate in peripheral blood were hCD45^+^ and randomly assigned into experimental groups. We performed genotyping of the *HLA* locus using PCR-specific oligonucleotide probing and PCR-sequence-based typing^61^. HLA types are presented in Supplementary Table 1. All mice were treated according to Institutional Animal Care and Research Committee-approved protocols.

### Plasmids, hydrodynamic injection and HIV-1 infection of humanized mice

Human EF1α/HTLV hybrid promoter-driven IL-21-encoding pIL21 or control pORF (Invivogen) were grown in bacteria and purified using NucleoBond PC2000 endotoxin-free plasmid DNA isolation kit (Macherey-Nagel). Before use, each plasmid batch was tested for cytokine expression by transfection into HEK293T cells and culture supernatants were assayed for IL-21 by enzyme-linked immunosorbent assay (ELISA). Hydrodynamic injection was performed as previously described^62^,^63^. Humanized mice were first pre-warmed with a heating lamp in sterile microisolator cages inside a laminar flow cabinet. Mice were injected in the tail vein with 10 μg plasmid resuspended in 2 ml (∼10% body weight) PBS within 10 s. Three days after hydrodynamic injection of plasmid, mice were bled and assayed for plasma cytokine expression. At this time point, all animals with detectable cytokine expression were then infected i.p. with10^5^ TCID_50_ of HIV-1_JR-CSF_. This dose was pre-tested to achieve 100% infection rate after i.p. injection of the virus supernatant. Venous blood was obtained by submandicular puncture and centrifuged at 2,000 r.p.m. for 5 min to isolate plasma (stored at −80 ^o^C until use). Plasma viral RNA was isolated from blood with the QIAamp Viral RNA Mini Kit (Qiagen) and HIV-1 RNA were determined by quantitative reverse transcription–PCR with the QuantiFast SYBR Green RT-PCR kit (Qiagen) as described^58^.

### Cell isolation and culture

Single cell suspension of splenocytes were prepared by mechanical disruption of organs and clarified by passing the cell suspension through 70 μm strainers. Splenocytes were treated with ACK lysis buffer (GIBCO) for 5 min to lyse red cells, washed and resuspended in culture medium for use. Fresh PBMCs were isolated from whole blood or buffy coat by Ficoll-Hypaque (GE Healthcare) and frozen PBMCs were thawed and recovered overnight in complete medium (see below) before subsequent cell isolation. Total CD4 T cells were isolated by negative selection using human CD4 negative selection kit II (Miltenyi Biotec, #130-096-533). For isolation of human CD4 T cells from BLT splenocytes, we used biotinylated anti-mouse CD45 (30-F11, eBioscience) antibody (5 μg per 10 × 10^6^ cells) and anti-biotin microbeads (Miltenyi) to first deplete mouse lymphocytes before CD4 negative selection yielding >90% CD3^+^CD4^+^ T cells. In all cases, cell suspensions were cultured at 37 ^o^C in 5% CO_2_ in complete medium (RPMI medium supplemented with 2 mM L-glutamine, 1 mM sodium pyruvate, 10 mM HEPES, nonessential amino acids, 50 IU ml^−1^ penicillin, 50 μg ml^−1^ streptomycin (Life Technologies) and 10% foetal bovine serum). To determine microRNA species expression after cytokine treatment, 0.5–1 × 10^6^ purified total CD4 T cells were stimulated overnight (16–18 h) at 37 ^o^C with 25 ng ml^−1^ of IL-17A, IL-21, IL-23, IFNγ or IFNα where indicated in 24-well tissue culture plates. In some experiments, CD4 T cells were stimulated with plate-bound anti-CD3 (5 μg ml^−1^) and anti-CD28 (5 μg ml^−1^) overnight (16–18 h) at 37 ^o^C in the presence and absence of IL-21. Subsequently, stimulated cells were washed with PBS and lysed with the Qiazol reagent protocol (Qiagen) or the miRNeasy Mini Kit (Qiagen) and subjected to total RNA isolation as described below.

### Generation of microRNA and miR-ZIP lentiviruses

For microRNA generation, pre-microRNA genomic regions were PCR amplified from total human DNA using the following primers (note: miRNA-specific sequences are in upper case and restriction sites are in bold) hsa-miR-29: hsa-miR-29bF-NotI: 5′-ctgacct**gcggccgc**CAGGCATGCTCTCCCATC-3′ and hsa-miR-29bR-BamHI: 5′-ctgacct**ggatcc**GTCAGCTACATGTGAGGCAGG-3′; hsa-miR-297: hsa-miR-297F-NotI: 5′-gatgat**gcggccgc**TACTCTGATTCCCTTTTCCC-3′ and hsa-miR-297R-BamHI: 5′-gatgat**ggatcc**GGTCTGTCGTTGACTGGAAC-3′. Amplified fragments were independently subcloned into the lentiviral plasmid vector *U6*-promoter pHAGE-U6 (Harvard Plasmid Core) via BamHI and NotI digestion. All positive clones were confirmed by sequencing. For miR-ZIP generation, asymmetric shRNAs were designed with the following rules and subcloned into the pLKO.1 lentiviral vector using AgeI and EcoRI digestion as described in the RNAi Consortium website (http://www.broadinstitute.org/rnai/public/). Forward: 5′-CCGG–mutated mature miRNA–CTCGAG–reverse complement of mature miRNA–TTTTTG-3′. Reverse: 5′-AATTC–reverse complement of forward oligo (starting from polyT and excluding CCGG)-3′. The oligonucleotides utilized to target miR-29b were: 29bZIP-F: 5′-CCGGTAGCACCTTTAGAAATCATTG-CTCTCGAGAACACTGATTTCAAATGGTGCTATTTTTG-3′; 29bZIP-R: 5′-AATTCAAAA-ATAGCACCATTTGAAATCAGTGTTCTCGAGA-GCAATGATTTCTAAAGGTGCTA-3′. Positive clones were confirmed by restriction digestion and sequencing. Lentiviruses were produced in 293T cells by co-transfecting U6-promoter pHAGE-based vectors and plasmids encoding gag/pol and VSVg, following standard methods. Supernatants were collected 2 days after transfection and viruses were concentrated via ultracentrifugation.

### HIV-1 infection of T-cell lines

Jurkat cells were transduced with lentivirus supernatants (1:50) by centrifugation (2 h at 1,000*g*) in the presence of polybrene (8 μg ml^−1^). Transduction efficiency (∼90%) was analysed by flow cytometry on the basis of GFP expression after 48 h. Transduced cells were infected with NL4-3-HIV-1-Luc^24^ and assessed for luciferase activity after 72 h. CEM-GXR25 T cell clones were infected with miR-ZIP lentiviruses for 48 h by centrifugation (2 h at 1,000*g*) in the presence of polybrene (8 μg ml^−1^ final concentration). Transduced CEM-GXR25 cells were selected with 1 μg ml^−1^ puromycin for 2 weeks before HIV-1 infection. HIV-1 infection in CEM-GXR25 cells was determined by the frequency of GFP^+^ cells after 72 h (ref. 25).

### Antibodies and flow cytometry

Cells (0.5–2 × 10^6^) were washed with staining buffer (PBS plus 5% FBS) and surface antigens were stained with fluorochrome-conjugated antibodies for 45 min at 4 ^o^C. Antibodies were used at a 1:200 dilution in a final volume of 200 μl. Fluorochrome-conjugated murine (m) and human (h) specific antibodies used were obtained from Biolegend (mCD45, 30-F11; hCD4, OKT4; hCD8α, HIT8a; hCD45, HI30; hIL-17A, BL168; hIFNγ, 4S.B3; CD56, HCD56; hCD45RO, UCHL1; hCCR5, HEK/1/85a; hCXCR4, 12G5; hCD3; OKT3), Life Technologies (hCD3 PE-Texas Red, MCHD0317) and eBiosciences (CD317/tetherin/BST2, 26F8; Ki-67, 20Raj1). All HIV-infected samples were fixed with 4% paraformaldehyde in PBS for virus inactivation. Where indicated, intracellular cytokines were stained using the Foxp3 staining kit protocol (eBiosciences) after cell suspensions were stimulated for 5 h with 50 ng ml^−1^ phorbol-12-myristate-13-acetate (Calbiochem) and 1 μM ionomycin (Calbiochem) in the presence of 1 × GolgiStop (BD Biosciences) at 37 ^o^C. Dead cells were excluded by staining with Live/Dead fixable Yellow or Violet dye (Life Technologies). Samples were acquired on an LSRII or LSRFortessa (BD Biosciences) instrument. Flow cytometer data were analysed using FlowJo (TreeStar).

### RNA isolation and quantitative real-time PCR

Total RNA was isolated from cells using the Qiazol reagent protocol or the miRNeasy Mini Kit to enrich for miRNA. Purified RNA were treated with DNase I reagent (Ambion) to further eliminate any genomic DNA contamination before use. For protein coding genes, HIV-1 complementary DNA (cDNA) and pri-miRNA quantification, 100–500 ng total RNA was reverse transcribed to cDNA using a high capacity cDNA reverse transcription kit with random priming (Applied Biosystems, #4368814). Mature miRNA expression was quantified using stem-loop miRNA-specific PCR assays^64^. For microRNA quantification, 5–10 ng total RNA was reversed transcribed to cDNA with microRNAs species-specific primers (Applied Biosystems, #4366596). TaqMan (Life Technologies/Applied Biosystems) gene or microRNAs expression PCR assays are listed in Supplementary Table 2. Gene expression levels were calculated based on the change in cycling threshold (**Δ***C*_T_) method as 2^−**Δ**CT^, where **Δ***C*_T_ is (*C*_T_(gene-of-interest)−*C*_T_(‘housekeeping’gene)). Protein coding genes and pri-miRNA were normalized to *GAPDH* or *ACTB* while miRNA expression was normalized to *U6* transcripts.

### Quantification of HIV-1 mRNA and DNA

HIV-1 mRNA and DNA were quantified as described^65^,^66^. For HIV-1 mRNA quantification, DNase I-treated RNA isolated from purified CD4 T cells were reverse transcribed into cDNA and quantified using TaqMan HIV-1-specific probe (Supplementary Table 2). HIV-1 mRNA expression were normalized to *ACTB* in the same samples. HIV-1 late RT and integrated DNA were assessed in total DNA that was isolated from purified CD4 T cells. Quantitative PCR were performed using the following primers: HIV-1 late RT, MH531 (forward): 5′-TGTGTGCCCGTCTGTTGTGT-3′; late RT, MH532 (reverse): 5′-GAGTCCTGCGTCGAGAGAGC-3′; late RT probe: 5′-(FAM)-CAGTGGCGCCCGAACAGGGA-(TAMRA)-3′; integrated HIV-1, MH535 (forward): 5′-AACTAGGGAACCCACTGCTTAAG-3′, Alu primer SB704 (reverse): 5′-TGCTGGGATTACAGGCGTGAG-3′, Alu probe MH603: 5′-(FAM)-ACACTACTTGAAGCACTCAAGGCAAGCTTT-(TAMRA)-3′. Amplification of SOCS3 (exon 1 primers, Supplementary Table 3) was used for the quantification and normalization of input cell DNA. Serial dilutions of DNA from cell lysates of HIV-1-infected 293T cells (provided by Dr F. Bushman, University of Pennsylvania, USA) were used as references and positive controls.

### ELISA

Human IL-21 protein was determined in BLT mice plasma or culture supernatant using the human IL-21 ELISA MAX Deluxe Set (Biolegend; #433804). HIV-1 p24 antigen in HLAC culture supernatants was determined using the Alliance HIV-1 p24 ELISA kit (NEK050001; Perkin Elmer). Samples were assayed in duplicates and concentrations determined off of a standard curve.

### Western blotting

Purified splenic CD4 T cells were treated for 16–18 h with the indicated cytokines and lysed in ice-cold RIPA buffer (Pierce) supplemented with protease inhibitors (Roche). Lysates were separated by SDS–PAGE electrophoresis, transferred onto polyvinylidene difluoride membranes and probed with antibodies against SAMHD1 (1 μg ml^−1^, SAB2102077; Sigma), APOBEC3G (5 μg ml^−1^, PRS3257; Sigma) and β-Actin (1:1000, 13E5; Cell Signaling). Original images of western blots shown in Supplementary Fig. 4b are included as a separate Supplementary Fig. 11.

### ChIP assay

ChIP assays were done as described^67^ on purified human splenic CD4 T cells stimulated with mIL-21 (R&D Systems) for 6 h. After stimulation, cells were cross-linked for 10 min at 25 °C with 1% (vol/vol) formaldehyde, and the formaldehyde was then inactivated by the addition of 125 mM glycine. Chromatin extracts containing DNA fragments with an average size of 500 bp were immunoprecipitated with 5 μg of anti-STAT3 (C-20, Santa Cruz Biotechnology) antibody or rabbit IgG per ∼1 ng ChIP DNA input. ChIP experiments were performed on CD4 T cells pooled from three donors to obtain sufficient material. Each primer amplified a single product of the correct size, as confirmed by dissociation-curve analysis. Fold enrichment was calculated relative to STAT3 binding in the promoter region (P3) of *MIR29B1/29A* (Fig. 2d). ChIP primers are listed in Supplementary Table 3.

### Statistical analysis

All statistical analyses were performed using GraphPad Prism or MS Excel.

## Additional information

**How to cite this article:** Stanley, A. *et al*. IL-21 induces antiviral microRNA-29 in CD4 T cells to limit HIV-1 infection. *Nat. Commun.* 6:7562 doi: 10.1038/ncomms8562 (2015).

## Supplementary Material

Supplementary Information Supplementary Figures 1-11 and Supplementary Tables 1-3
