# Supplementary material for: IL-21 induces antiviral microRNA-29 in CD4 T cells to limit HIV-1 infection
Source: Nat Commun. 2015 Jun 25;6:7562. doi: 10.1038/ncomms8562 (PMC4481879; doi:10.1038/ncomms8562)
Supplement: Supplementary Information — Supplementary Figures 1-11 and Supplementary Tables 1-3 [file ncomms8562-s1.pdf]

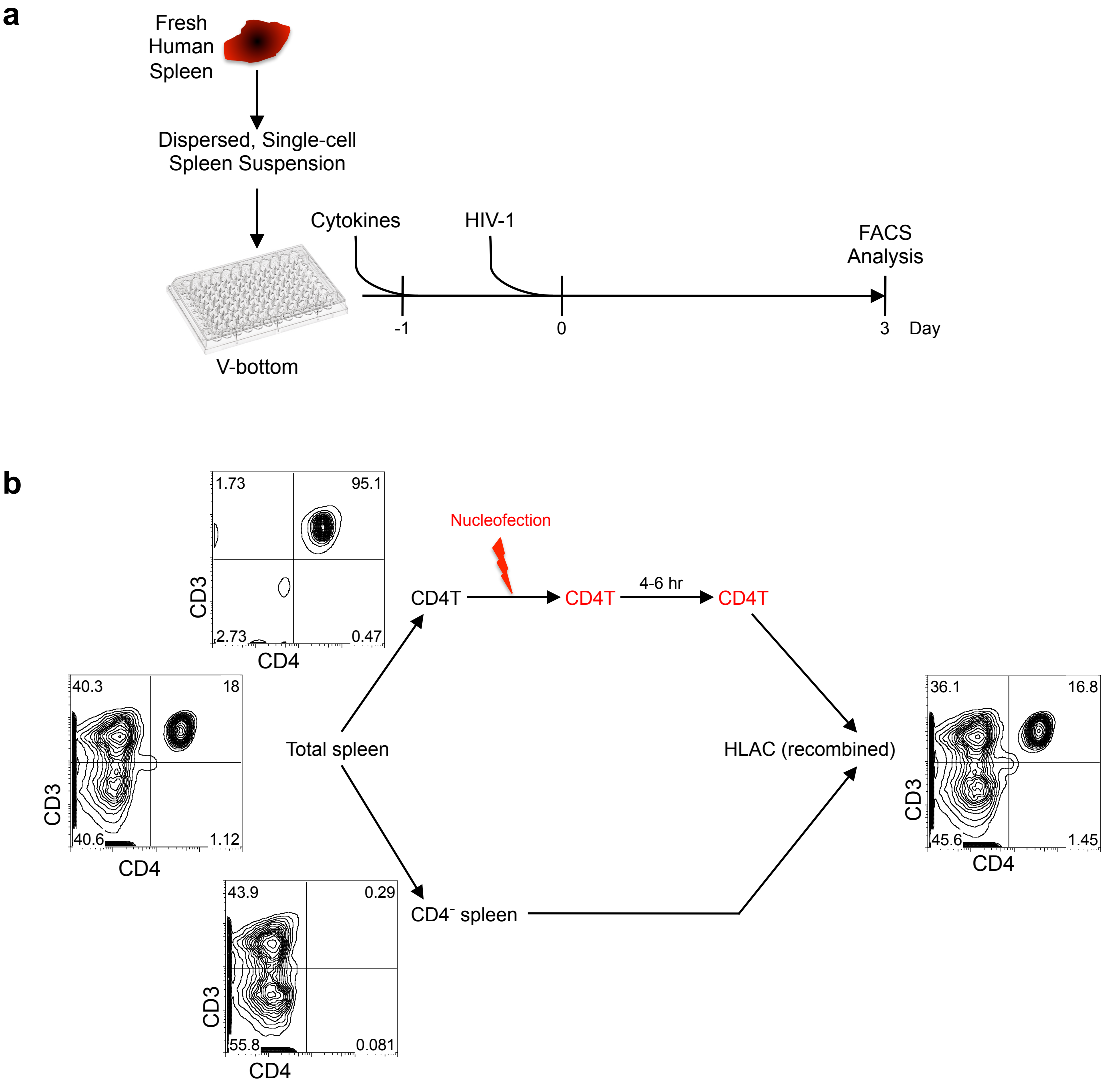

Supplementary Figure 1. Protocol for human lymphoid organ aggregate cultures (HLAC). (a) Freshly dissected human spleens were processed into single-cell suspensions, plated into 96-well v-bottom plates and treated with cytokines overnight before infection with GFP-tagged HIV-1 by centrifugation at 1200*g* at room temperature for 2 hrs. Except when otherwise indicated, infection was assessed after 72 hr by GFP expression using flow cytometry or p24 ELISA of culture supernatants. (b) Splenic HLAC protocol with microRNA predepletion. Total CD4 T cells were purified from spleen and nucleofected (Amaxa) with antagomir locked nucleic acid (LNA), rested and re-combined with CD4-depleted splenic fraction. The reconstituted splenic HLAC were then subjected to cytokine treatment and HIV-1 infection as in (a). Representative flow cytometric plots of CD3 versus CD4 at the different experimental steps are shown. Numbers indicate the frequency of cells in each gate.

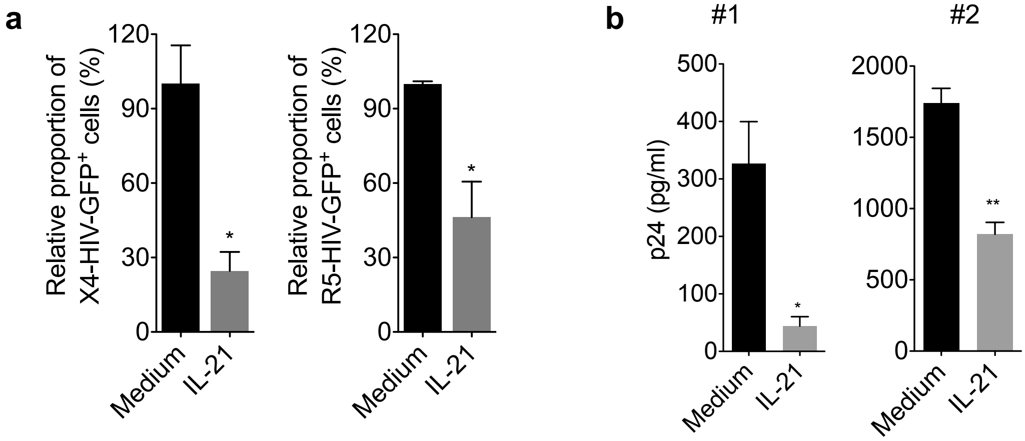

Supplementary Figure 2. IL-21-mediated HIV-1 suppression in human lymphoid organ aggregate cultures (HLAC). (**a**) Relative proportion of GFP$^+$ cells in untreated (medium, 100%) or IL-21 treated HLACs 72 hours post infection with X4-HIV-GFP or R5-HIV-GFP. GFP expression was determined in gated CD3$^+$ cells as shown in **Fig. 1a.** Data are representative of 2 (R5-HIV-GFP infection) to >10 donors (X4-GFP infection). **(b)** HIV-1 p24 protein in HLAC supernatants infected with X4-HIV-GFP from two representative donors. *, $p < 0.05$; **, $p < 0.005$; unpaired Students *t*-test.

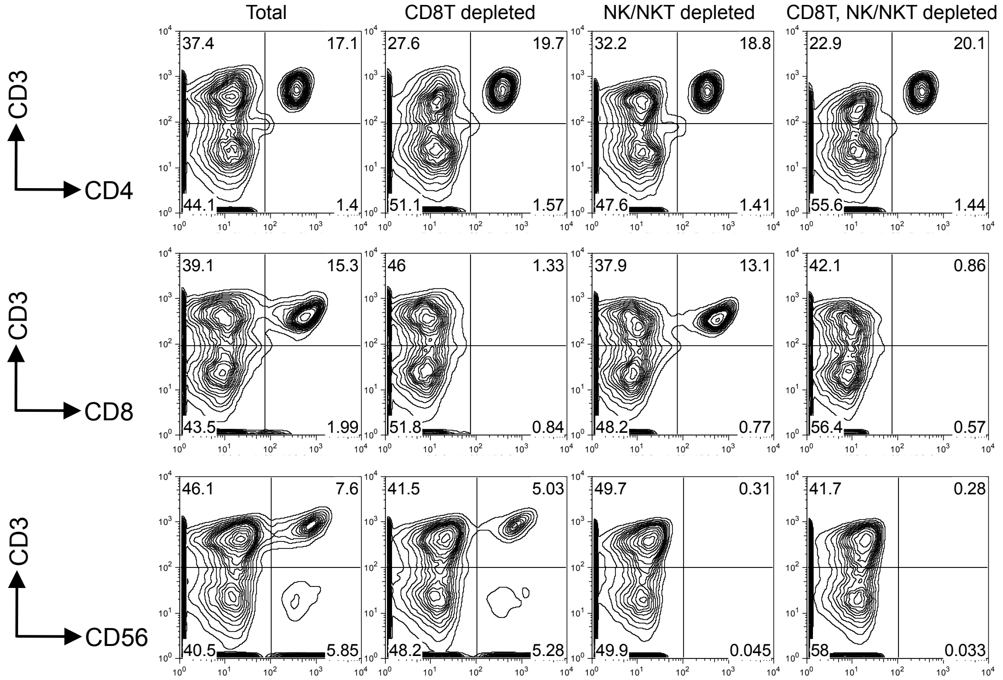

Supplementary Figure 3. Representative flow cytometry plots of CD3 versus CD4 (top), CD3 versus CD8 (middle), CD3 versus CD56 (bottom) in total HLACs or CD8 and NK/NKT cell depleted HLACs. Numbers indicate frequency of cells in each gate.

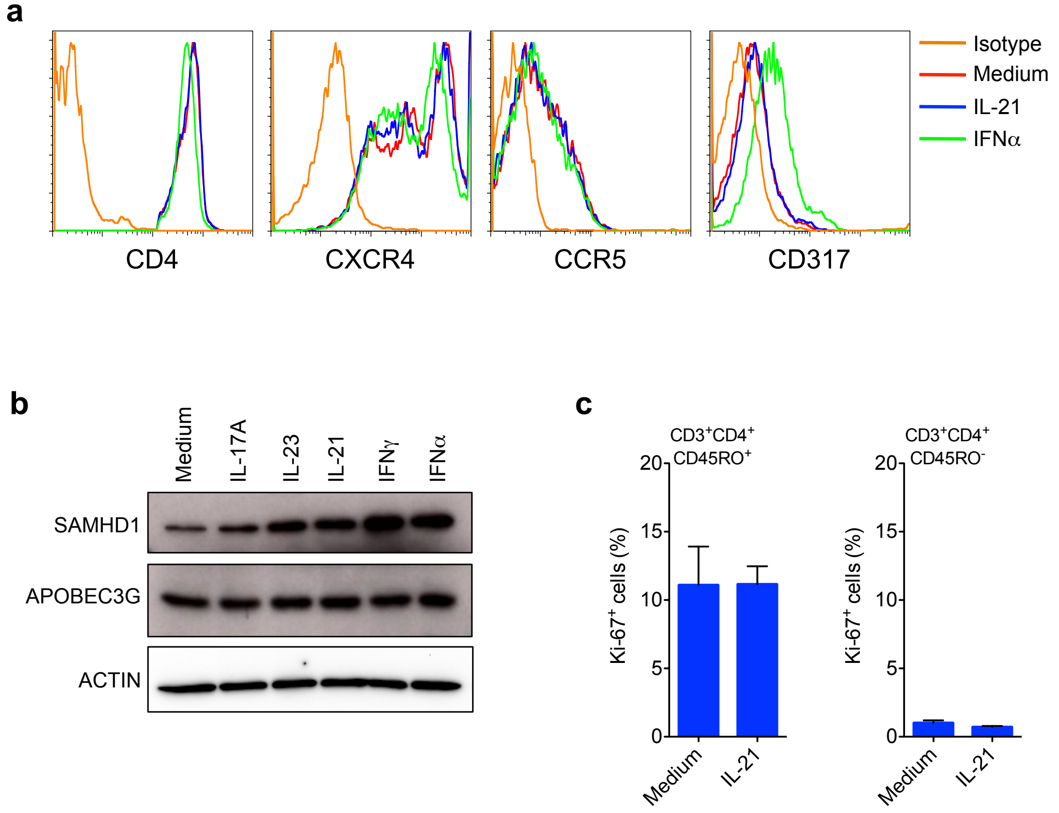

Supplementary Figure 4. Expression of HIV-1 entry coreceptors and restriction factors in cytokine treated human splenic CD4 T cells. (a) Flow cytometric histogram plots of CD4, CXCR4, CCR5 and CD317 (tetherin) expression on human splenic $CD4^{+}CD3^{+}$ cells after 16 hr treatment with the indicated cytokines. (b) Western blot for APOBEC3G, SAMHD1 and ACTIN on purified total human CD4 T cells treated for 18 hr with the indicated cytokines. Original western blots are shown in Supplementary Fig. 11. (c) Intracellular Ki-67 expression in human splenic CD4 T cells treated with IL-21 for 3 days. Data are average (± s.e.m.) of triplicate wells and representative of two independent donors.

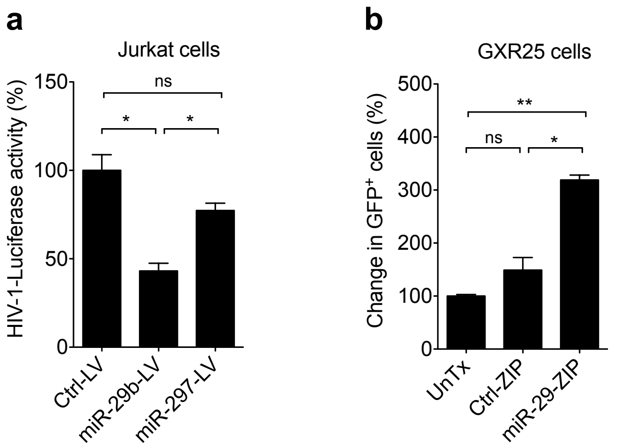

Supplemental Figure 5. MicroRNA-29 is a cell-intrinsic HIV-1 restriction factor in CD4 T cells. (**a**) Percent change in luciferase activity of lysates from Jurkat T cells stably transduced with control (Ctrl-LV), miR-29b-LV or miR-297-LV lentivirus and infected with HIV-1_{NL4-3}-luciferase for 72 hours; 100% = Ctrl-LV transduced cells. (**b**) Percent change in GFP^+ CEM-GXR25 T cells stably transfected with lentivirus encoding the indicated "miR-ZIPs" 72 hours post infection with HIV-1_{NL4-3}. Data are average (± s.e.m.) of triplicate wells and representative of two experiments. *, *p* <0.05; **, *p* <0.005; *ns*, not significant; unpaired Students *t*-test.

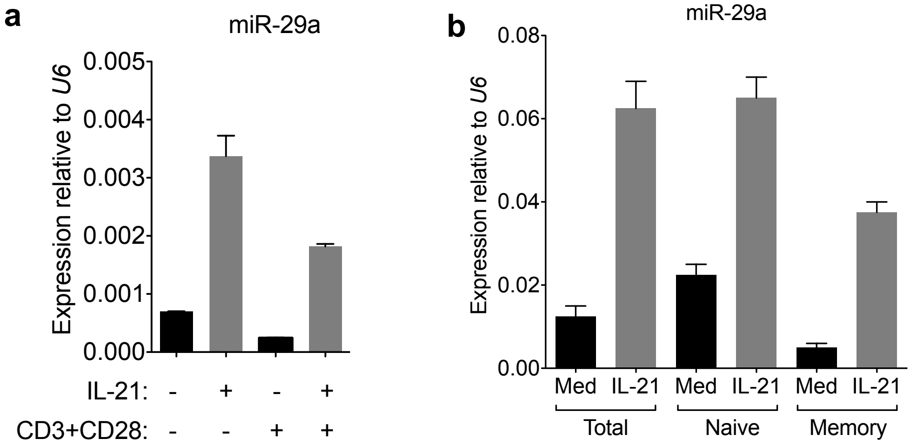

Supplementary Figure 6. Regulation of miR-29 in human splenic CD4 T cells. (a) Expression of miR-29a in activated CD4 T cells in the presence or absence of IL-21. Purified total CD4 T cells were stimulated with plate-bound anti-CD3 and anti-CD28 antibodies overnight for 16 hours and assessed for miR-29a expression. Data are average (± s.d.) of duplicate wells and representative of two donors. (b) Expression of miR-29 in subsets of CD4 T cells in the presence or absence of IL-21. Data are average (± s.d.) of duplicate wells.

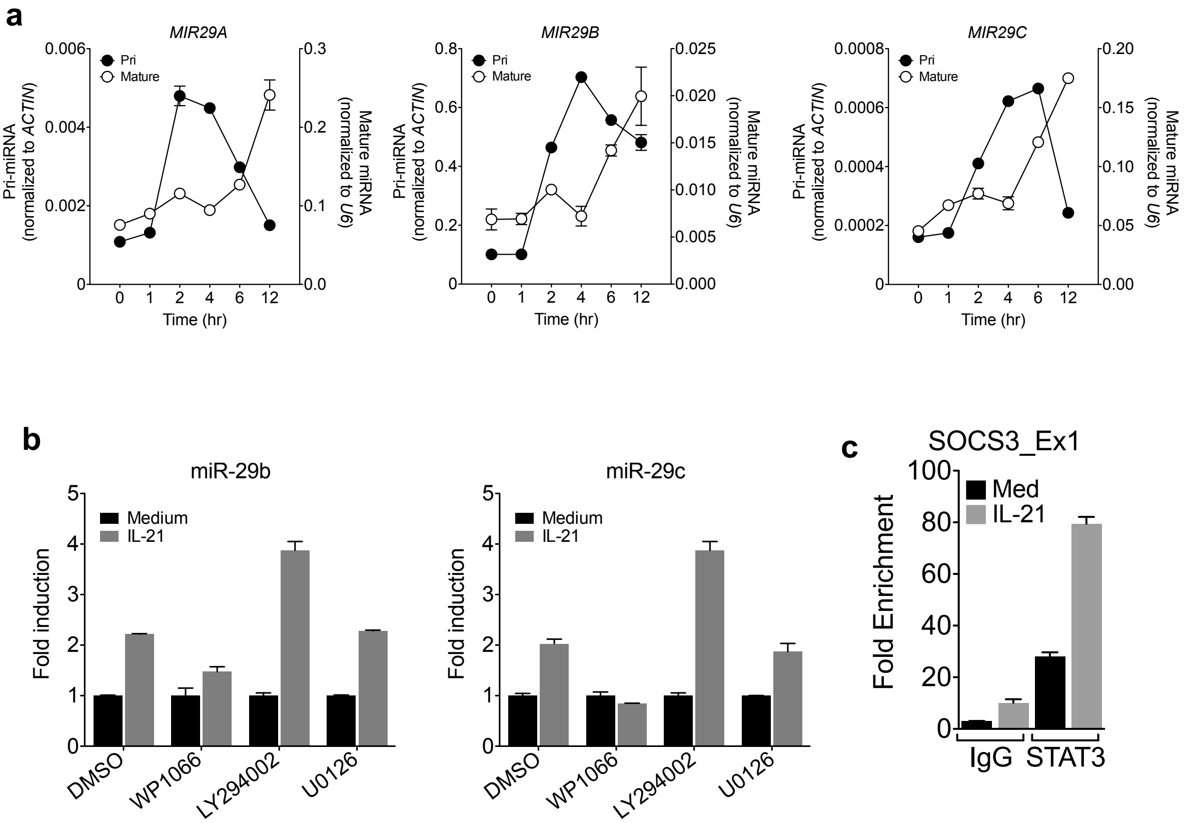

Supplementary Figure 7. Kinetics and mechanism of microRNA-29 gene regulation. (a) Kinetics of pri-miR-29 and mature miR-29 induction by IL-21. Data are actual expression levels (normalized to *ACTIN* or *U6* as indicated) of the data shown in Fig. 2b. (b) Fold induction of miR-29b and miR-29c species in human splenic CD4 T cells treated with IL-21 for 12 hours in the presence or absence of pharmacological inhibitors against STAT3 (WP1066), PI3-kinase (LY294002) and MEK (U1026). Data are average (± s.d.) of duplicate wells. (c) STAT3 binding to sequences upstream of *SOCS3* gene in untreated and IL-21 stimulated splenic CD4 T cells determined by ChIP assay.

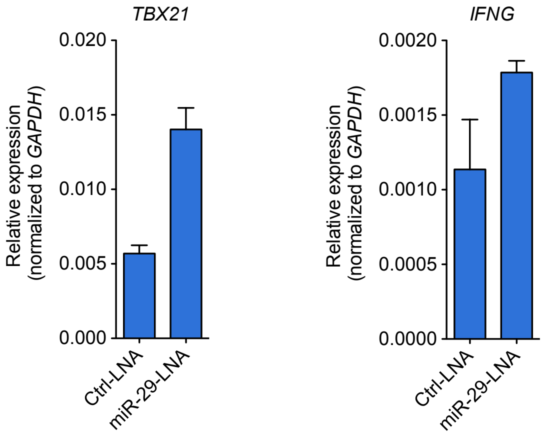

Supplementary Figure 8. T-bet (*TBX21*) and *IFNG* mRNA expression in human splenic CD4 T cells 48 hr after nucleofection with miR-29b-LNA antagomir. Data are average (± s.d.) of duplicate wells.

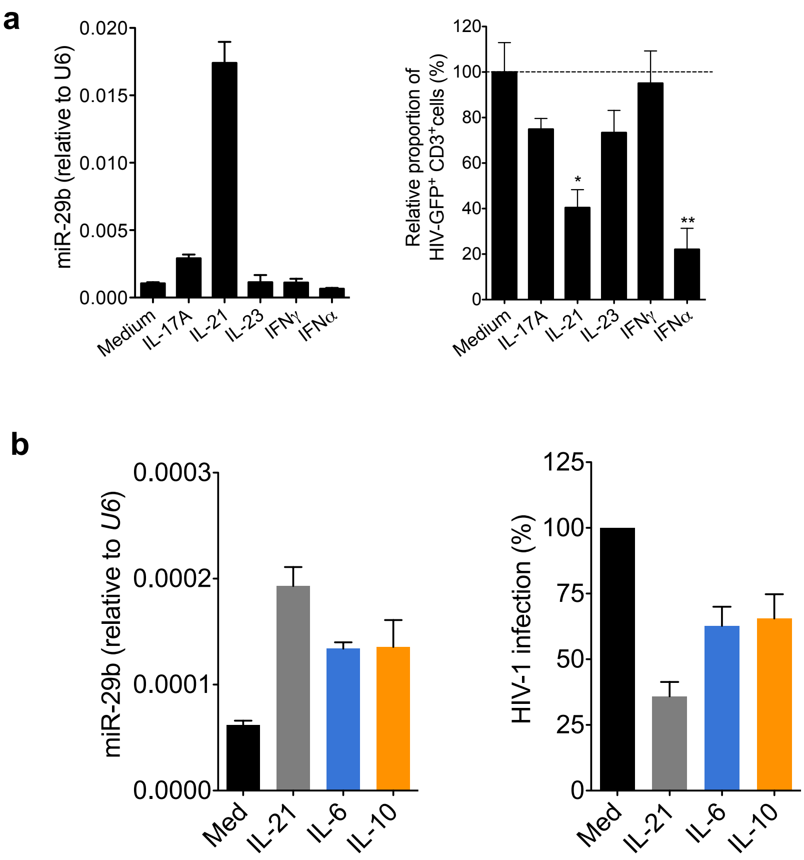

Supplementary Figure 9. Specificity of IL-21 antiviral activity. (**a**) Relative expression of miR-29b in purified human splenic CD4 T cells (left) after 12 hr and proportion of GFP^{+}CD3^{+} cells (average ± s.e.m. of triplicate wells) in HLACs infected with X4-HIV-GFP (normalized to medium alone cultures, right) after 72 hours of infection. (**b**) Relative expression of miR-29b after 12 hr (left) and HIV-1 infection in three donors in the presence of STAT3-activating cytokines (right) after 72 hours. Cytokines were used at 25 ng ml^{-1}. MicroRNA expression data are average (± s.e.m. of duplicate wells) and representative of two donors. *$p < 0.05$; **$p < 0.005$; Student's t-Test.

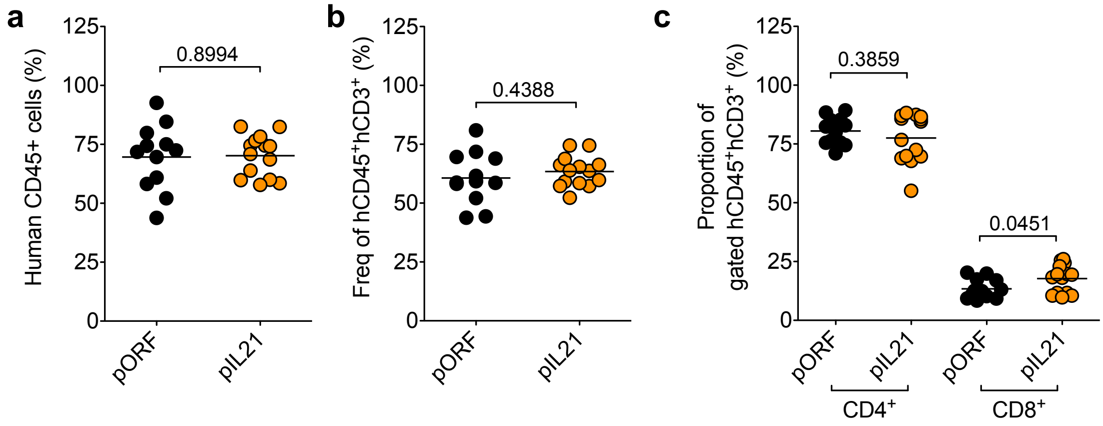

Supplementary Figure 10. Human immune cell reconstitution in peripheral blood of BLT humanized mice hydrodynamically injected with pORF (*n* = 12) and pIL21 (*n* = 14) plasmids at 2 weeks post infection. (a) Frequency of total human $CD45^{+}$ cells. (b) Frequency of human $CD3^{+}$ T cells within the $hCD45^{+}$ cells. (c) Frequency of human $CD4^{+}$ and $CD8^{+}$ T cells. Each data point represents one mouse; *p* values (Student's *t*-test) are indicated.

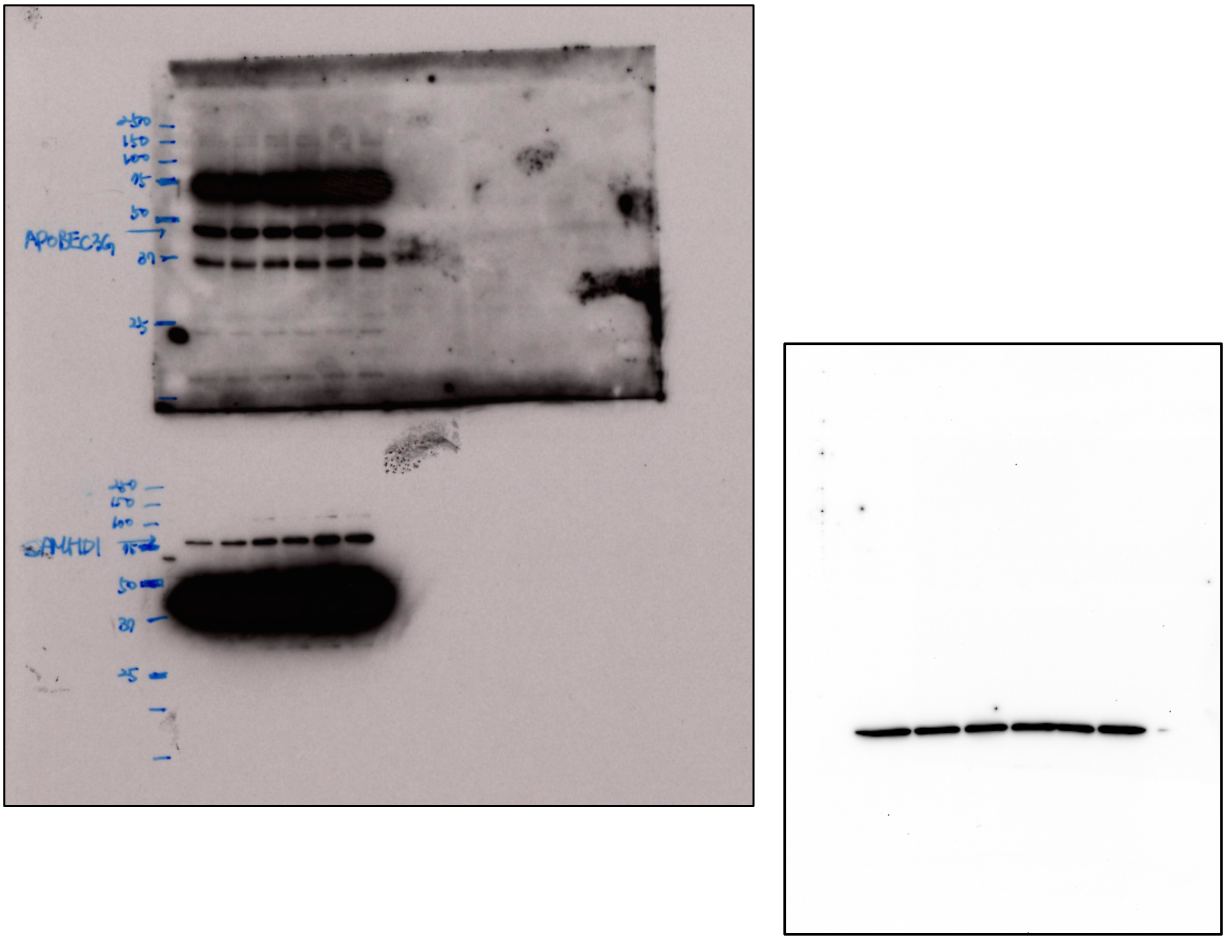

Supplementary Figure 11. Original images of western blots shown in Supplementary Fig. 4b. Left, APOBEC3G (~48 kDa) and SAMHD1 (~72 kDa); right, Actin. Lanes, left to right: 1, medium (untreated); 2, IL-17A; 3, IL-23; 4, IL-21; 5, IFNγ; 6, IFNα.

Supplementary Table 1. HLA haplotypes of donor tissues used for generating the BLT mice reported in this study

Batch	HLA-A1	HLA-A2	HLA-B1	HLA-B2	HLA-C1	HLA-C2
50	301	3101	3501	5101	401	1502
89	0301	0301	0702	3501	0401	0702

Supplementary Table 2. TaqMan qPCR primer/probe sets and assay ID

Primer/probe	Life Technologies Assay ID
IFNG	Hs00989291_m1
IL17A	Hs00174383_m1
IL21	Hs00222327_m1
TBX21	Hs00203436_m1
GAPDH	Hs02758991_g1
ACTB	Hs01060665_g1
hsa-miR-29a	002112
hsa-miR-29b	000413
hsa-miR-29c	000587
hsa-miR-142-5p	002248
U6 snRNA	001973
HIV1-LTR	Pa03453409_s1
pri-hsa-mir-29a	Hs03302672_pri
pri-hsa-mir-29b1	Hs03302748_pri
pri-hsa-mir-29c	Hs04225365_pri

Supplementary Table 3. ChIP primers

Primer	Sequence
SOCS3_Ex1_F	GAAGTCTCCGTCCTTGGGGCTGAGCC
SOCS3_Ex1_R	CAGGGGAAGCTCGAGGGACGCGCGC
29b1/29a_P3_F	TCTTTTCATAAGGAGATCCCAG
29b1/29a_P3_R	ATGTGCCTTTCTCTGCAGTCAACAA
29b1/29a_P12_F	TGTG TCCTGCTGGTGGAGAGCACAA
29b1/29a_P12_R	CCTGCCACATCTGGCATGATTTTACC
29b1/29a_P14_F	AGCTGCCTCTTCCAGCGGGAGGCT
29b1/29a_P14_R	ACGGAACCAGGGAGGCTTTGGTCA
29b2/29c_P4_F	CTAACCCTGCTTGCTTGTGCTGCGCTACCA
29b2/29c_P4_R	ATCTGGCTGGAACACACATCAGTCACGTAA
29b2/29c_P9_F	GAGCCAAAGAGAGTTTCTATTACTGTGGG
29b2/29c_P9_R	GGCAGAATGGGCAGCTGAAGTGGTCG
